# Associations of fibroblast growth factor 21 with renal parameter changes during pemafibrate treatment in patients with type 2 diabetes

**DOI:** 10.1007/s13340-026-00945-y

**Published:** 2026-11-10

**Authors:** Hideyuki Okuma

**Affiliations:** Department of Diabetes and Endocrinology, Nerima Hikarigaoka Hospital, 2-5-1 Hikarigaoka, Nerima-Ku, Tokyo, 1790072 Japan

**Keywords:** Pemafibrate, Diabetic kidney disease, Type 2 diabetes mellitus, Fibroblast growth factor 21

## Abstract

**Background:**

Experimental studies suggest that fibroblast growth factor 21 (FGF21) is involved in pathways related to peroxisome proliferator-activated receptor alpha (PPARα) activation, whereas elevated circulating FGF21 has also been associated with metabolic stress and kidney dysfunction. We examined the associations of FGF21 with renal parameter changes following the initiation of pemafibrate, a selective PPARα modulator, in patients with type 2 diabetes mellitus (T2DM).

**Methods:**

In this retrospective study using prospectively collected blood samples, 81 patients with T2DM who initiated pemafibrate and continued treatment for ≥ 2 years were analyzed. Clinical data and serum high-sensitivity C-reactive protein (hsCRP) and FGF21 levels were assessed at baseline and two years. The main outcomes were changes in estimated glomerular filtration rate (eGFR) and log-transformed urinary albumin-to-creatinine ratio (UACR). Multivariable linear regression analyses were performed.

**Results:**

During the two-year observation period, triglyceride, hsCRP, and UACR levels decreased, whereas FGF21 increased; eGFR remained stable. The change in eGFR was more favorable in patients with baseline eGFR of 30 to < 60 mL/min/1.73 m^2^ than in those with eGFR ≥ 60 mL/min/1.73 m^2^. Higher baseline FGF21 and greater increases in FGF21 remained associated with less favorable changes in eGFR and UACR in multivariable analyses.

**Conclusions:**

Higher baseline FGF21 and greater increases in FGF21 were associated with less favorable renal parameter changes after pemafibrate initiation. Because this observational study lacked a control group, causal effects cannot be inferred. Prospective controlled studies are needed to determine the clinical significance of circulating FGF21.

**Supplementary Information:**

The online version contains supplementary material available at https://doi.org/10.1007/s13340-026-00945-y.

## Introduction

Diabetic kidney disease (DKD) is a major microvascular complication of diabetes and remains a leading cause of chronic kidney disease (CKD) and end-stage kidney disease worldwide [1, 2]. Despite advances in glycemic and blood pressure management, many patients with DKD continue to experience progressive kidney function decline, reflecting the complex and multifactorial pathophysiology of the disease [3]. Although sodium–glucose cotransporter 2 (SGLT2) inhibitors, glucagon-like peptide − 1 receptor agonists (GLP-1RAs), angiotensin receptor blockers (ARBs), and mineralocorticoid receptor antagonists (MRAs) have demonstrated renoprotective benefits, substantial residual renal risk remains, highlighting the need for additional therapeutic approaches and biomarkers reflecting renal risk [4, 5].

Peroxisome proliferator-activated receptor alpha (PPARα), a nuclear receptor highly expressed in renal proximal tubular cells [6], regulates fatty acid oxidation, energy metabolism, and inflammatory pathways. Experimental studies have shown that pharmacological activation of PPARα reduces albuminuria, renal inflammation, and fibrosis in diabetic models [7]. Although the use of the conventional PPARα agonist fenofibrate is limited in patients with moderate to severe renal impairment [8], pemafibrate is a selective PPARα modulator with high receptor selectivity and a favorable pharmacokinetic profile [9, 10]. Because pemafibrate is primarily metabolized in the liver and minimally excreted by the kidneys, it can also be used in patients with impaired renal function [11]. Clinical studies have reported changes in lipid and renal parameters during pemafibrate treatment in patients with diabetes and CKD [12], while experimental studies have demonstrated effects on renal inflammation, fibrosis, and fatty acid metabolism [13, 14].

Fibroblast growth factor 21 (FGF21) is a metabolic hormone whose circulating concentration is increased by PPARα activation. Experimental studies have suggested that FGF21 may participate in pathways involved in the renoprotective effects of fibrates, including modulation of oxidative stress and inflammation [15, 16]. However, the biological actions of FGF21 demonstrated experimentally should be distinguished from the clinical significance of circulating FGF21 concentrations. In patients with obesity, T2DM, and CKD, elevated circulating FGF21 levels have been associated with metabolic dysfunction and adverse renal outcomes [17–21], possibly representing a compensatory response to metabolic stress or impaired FGF21 responsiveness. Thus, whether circulating FGF21 levels during pemafibrate treatment reflect favorable biological responses, underlying metabolic stress, or other pathophysiological processes remains uncertain.

Therefore, the present study examined longitudinal changes in renal parameters and circulating FGF21 levels during the two years after pemafibrate initiation in patients with T2DM and evaluated the associations of baseline FGF21 levels and their longitudinal changes with changes in eGFR and UACR.

## Materials and methods

### Study design and participants

**Fig. 1:**
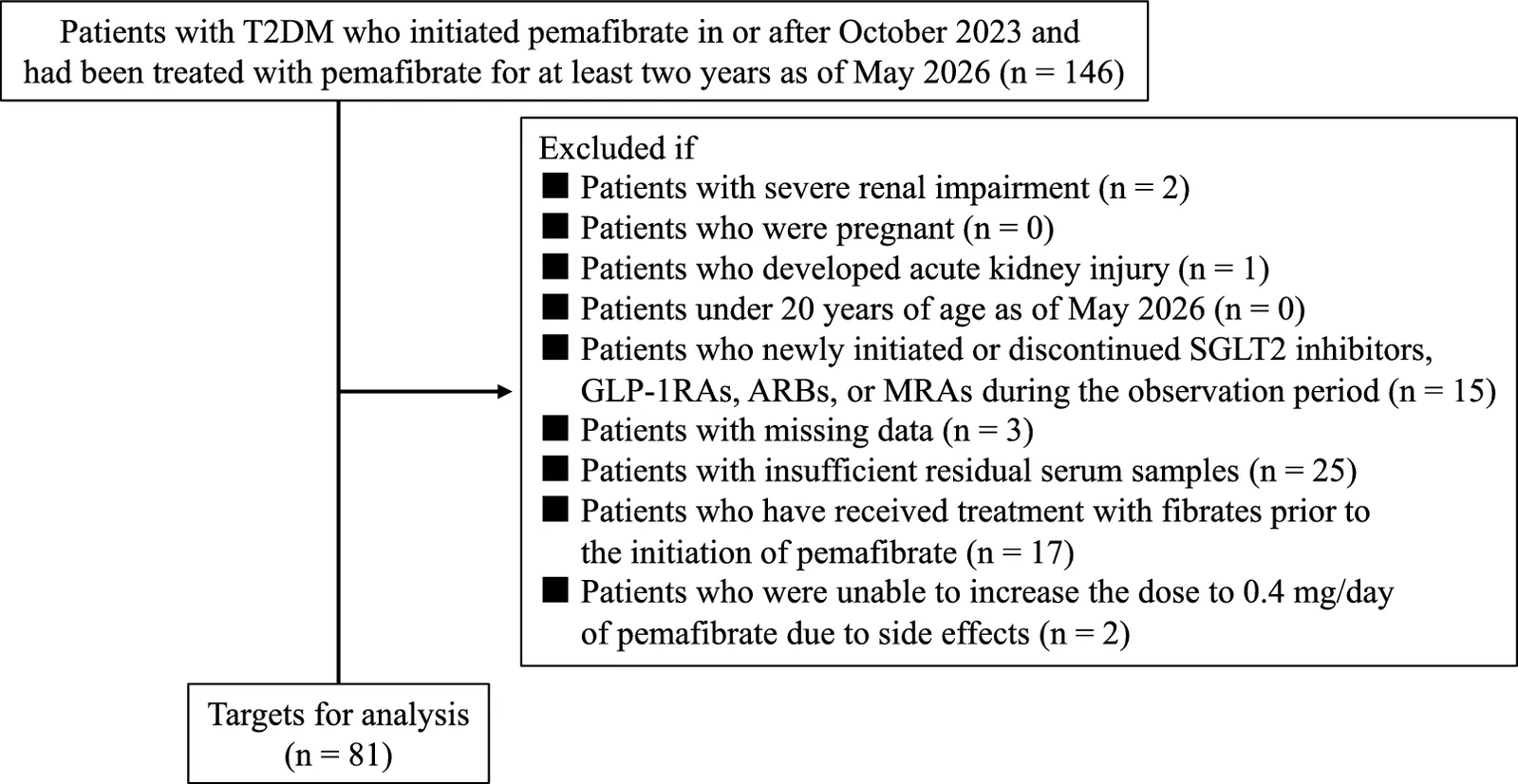
Flowchart showing the participant selection process. The process of selecting study participants is shown. ARBs, angiotensin receptor blockers; GLP-1 RAs, glucagon-like peptide − 1 receptor agonists; MRAs, mineralocorticoid receptor antagonists; SGLT2, sodium-glucose cotransporter 2; T2DM, type 2 diabetes mellitus

In this single-center retrospective observational study using prospectively collected peripheral blood samples, we enrolled patients with T2DM who visited Nerima Hikarigaoka Hospital from October 2023 to May 2026. All consecutive patients with T2DM who regularly attended the outpatient internal medicine clinic at Nerima Hikarigaoka Hospital during the study period were considered eligible for this study. The inclusion criteria were as follows: patients were not receiving fibrates at baseline; pemafibrate was initiated at 0.2 mg/day in or after October 2023 at our hospital; the dose was increased to 0.4 mg/day within two months of initiation and maintained at that dose thereafter; and patients had been treated with pemafibrate at our hospital for a total of at least two years as of May 2026. The last inclusion criterion was established due to findings indicating that accurate assessments of individual rates of kidney function deterioration are generally insufficient in instances where the follow-up period is less than two years [22]. During the process of recruiting research participants (Fig. 1), we excluded the following patients with T2DM: (1) those with severe renal impairment (undergoing renal replacement therapy or an eGFR of < 15 mL/min/1.73 m^2^), (2) those who were pregnant, (3) those who developed acute kidney injury as defined by the Kidney Disease Improving Global Outcomes (KDIGO) [23], (4) those under 20 years of age as of May 2026, (5) those who newly initiated or discontinued SGLT2 inhibitors, GLP-1RAs, ARBs, or MRAs during the observation period, (6) those with missing data, (7) those with insufficient residual serum samples. A total of 146 patients with T2DM initiated pemafibrate in or after October 2023 and had been treated with pemafibrate for at least 2 years as of May 2026. After excluding those who did not meet the aforementioned inclusion criteria or who met the exclusion criteria, 81 patients were included in the analysis (Fig. 1). To assess potential selection bias, available baseline characteristics were additionally compared between patients included in the analysis and eligible patients who were excluded from the analysis (Supplementary Table S1). In this study, we examined renal parameter changes over a 2-year period in these patients after pemafibrate initiation. The prospective collection and analysis of peripheral blood samples were approved by the institutional review board of Nerima Hikarigaoka Hospital. All participants provided written informed consent for the collection and analysis of peripheral blood samples. The retrospective analysis of clinical and laboratory data related to pemafibrate treatment was separately approved by the institutional review board of Nerima Hikarigaoka Hospital. For the retrospective analysis of clinical data, the requirement for additional written or oral informed consent was waived by the institutional review board, and information regarding the study was disclosed through an opt-out approach.

### Clinical data collection

**Fig. 2:**
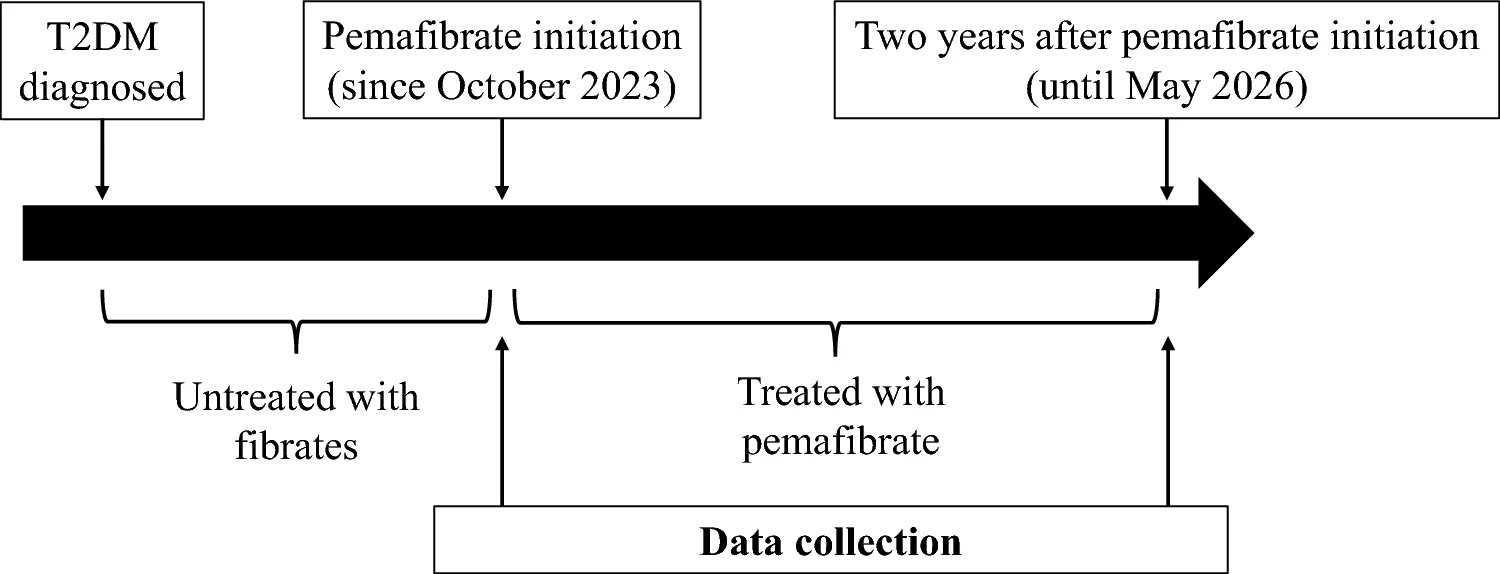
Timeline of this study. T2DM, type 2 diabetes mellitus

Figure 2 shows the timeline of this study. Data were collected at the initiation of pemafibrate and two years later. Specifically, data on demographics (including age, sex, duration of DM, smoking status, and body mass index [BMI]), physiological measurements (covering systolic and diastolic blood pressure [SBP and DBP]), complications of DM (such as diabetic neuropathy, retinopathy, DKD, and atherosclerotic diseases), laboratory test outcomes, and prescribed medications were collected. As part of their insurance-covered medical care, patients with T2DM had 9 parameters measured including hemoglobin A1c (HbA1c), triglycerides (TG), high-density lipoprotein cholesterol (HDL-C), low-density lipoprotein cholesterol (LDL-C), aspartate transaminase (AST), alanine transaminase (ALT), uric acid (UA), urinary albumin-to-creatinine ratio (UACR), and eGFR. Furthermore, at the same time, we collected residual blood samples to measure serum high-sensitivity C-reactive protein (hsCRP) and FGF21 levels. Data regarding these biochemical parameters were obtained from all participants. Blood samples were collected in the morning after fasting for at least 12 h. The levels of HbA1c were measured using latex agglutination. The eGFR was calculated using the CKD-EPI equation modified with a Japanese coefficient [24]. The concentrations of urinary albumin and creatinine were measured through turbidimetric immunoassay and enzymatic methods, respectively, and the UACR (mg/gCr) was calculated to evaluate albuminuria in spot urine samples. Serum hsCRP levels were quantified using the immunoturbidimetric CRP-Latex assay (Kamiya Biomedical Co., WA, USA), according to the manufacturer’s protocol. Serum FGF21 levels were measured using an enzyme-linked immunosorbent assay kit (Phoenix Pharmaceuticals, Inc., Burlingame, CA, USA), according to the manufacturer’s protocol. Measurements of serum hsCRP and FGF21 levels were performed in duplicate, with any discrepancies outside of three standard deviations prompted reanalysis. DKD was defined as diabetes with a UACR of ≥ 30 mg/g and/or an eGFR of < 60 mL/min/1.73 m^2^ in the absence of signs or symptoms of other primary causes of kidney damage according to the KDIGO guidelines. The main outcomes were changes in eGFR and log-transformed UACR during the observation period.

### Statistical analyses

We used Prism 9 (GraphPad Software Inc., San Diego, CA, USA) for statistical analyses. Continuous variables with a normal distribution were analyzed using the *t*-test, whereas non-normally distributed variables were evaluated using the Mann–Whitney U test or Wilcoxon signed-rank test. Nominal variables were analyzed using the Chi-square test or Fisher’s exact test. Spearman’s rank correlation coefficient was employed for correlation analyses. In multivariate linear regression analysis, we adjusted for the following variables using a stepwise procedure in Model 1: age, female sex, duration of DM, current smoking status, BMI, complications of DM excluding DKD, physiological measurements, laboratory parameters, and medications. In multivariate analysis using the two-year change in eGFR as a dependent variable, in addition to the variables selected in Model 1, Model 2 included baseline UACR, duration of DM, and HbA1c as forced entry variables, and Model 3 included baseline UACR, TG, and change in log-transformed TG as forced entry variables. In multivariate analysis using the two-year change in log-transformed UACR as a dependent variable, in addition to the variables selected in Model 1, Model 2 included baseline SBP, eGFR, duration of DM, and HbA1c as forced entry variables, and Model 3 included baseline SBP, eGFR, TG, and change in log-transformed TG as forced entry variables. Continuous variables that were not normally distributed were log-transformed using the natural logarithm before being entered into these models. A two-sided *P*-value of < 0.05 was considered statistically significant. Because the subgroup, correlation, and multivariable analyses were exploratory, no formal adjustment for multiple comparisons was prespecified or applied.

## Results

A total of 81 patients with T2DM were included in the analysis. The available baseline characteristics were generally comparable between the patients included in the analysis and those who were excluded, with no statistically significant differences observed in any of the parameters evaluated (Supplementary Table S1). The characteristics of the included participants are shown in Table 1. The mean baseline eGFR was 58.9 ± 12.2 mL/min/1.73 m^2^, and the median UACR was 27.2 (11.4–41.6) mg/gCr. Fifty-seven patients (70%) met the definition of DKD at baseline. When stratified by baseline renal function, 42 patients had an eGFR of ≥ 60 mL/min/1.73 m^2^ and 39 had an eGFR of 30 to < 60 mL/min/1.73 m^2^. Baseline eGFR was lower, hsCRP was higher, and FGF21 levels tended to be higher in patients with an eGFR of 30 to < 60 mL/min/1.73 m^2^ than in those with an eGFR of ≥ 60 mL/min/1.73 m^2^, whereas other baseline clinical characteristics, laboratory parameters, and medication use were generally comparable between the two groups.

**Table 1:** Baseline characteristics of the included participants and comparison based on baseline eGFR

	Overall (*n* = 81)	eGFR ≥ 60 (*n* = 42)	30 ≤ eGFR < 60 (*n* = 39)	*P-*value
Clinical characteristics				
Age (years)	56.8 ± 9.8	58.3 ± 9.7	55.3 ± 9.8	0.166
Female sex, *n* (%)	41 (51)	22 (52)	19 (49)	0.825
Duration of DM (years)	11 (5–19)	11 (6–19)	11 (4–19)	0.677
Current Smoker (*n*, %)	26 (32)	15 (36)	11 (28)	0.487
BMI (kg/m^2^)	25.3 ± 3.2	24.8 ± 3.5	25.8 ± 2.8	0.169
Complications of DM (*n*, %)				
Neuropathy	42 (52)	21 (50)	21 (54)	0.825
Retinopathy	30 (37)	15 (36)	15 (38)	0.822
DKD	57 (70)	18 (43)	39 (100)	< 0.001
Atherosclerotic diseases	20 (25)	13 (31)	7 (18)	0.205
Classification based on eGFR (mL/min/1.73m^2^) or UACR (mg/gCr)				
eGFR ≥ 90	4 (5)	4 (10)	0 (0)	
60 ≤ eGFR < 90	38 (47)	38 (90)	0 (0)	
30 ≤ eGFR < 60	39 (48)	0 (0)	39 (100)	
eGFR < 30	0 (0)	0 (0)	0 (0)	
UACR < 30	44 (54)	24 (57)	20 (51)	0.372
30 ≤ UACR < 300	35 (43)	16 (38)	19 (49)	
UACR ≥ 300	2 (3)	2 (5)	0 (0)	
Physiological testing				
SBP (mmHg)	131 ± 10	130 ± 9	132 ± 10	0.490
DBP (mmHg)	70 ± 6	70 ± 6	70 ± 6	0.639
Laboratory parameters				
HbA1c (%)	8.10 (7.50–8.80)	8.05 (7.50–8.75)	8.20 (7.50–8.80)	0.787
TG (mg/dL)	260 (199–329)	255 (210–322)	303 (195–336)	0.667
HDL-C (mg/dL)	40 (34–46)	41 (34–45)	40 (35–46)	0.917
LDL-C (mg/dL)	108 (96–117)	102 (93–115)	111 (101–121)	0.088
AST (U/L)	28 (16–35)	29 (18–32)	27 (16–36)	0.580
ALT (U/L)	39 (29–45)	39 (35–44)	39 (28–46)	0.667
UA (mg/dL)	5.6 (4.1–6.7)	5.4 (3.9–6.9)	5.8 (4.3–6.5)	0.616
UACR (mg/gCr)	27.2 (11.4–41.6)	26.2 (12.3–37.1)	27.7 (10.4–45.8)	0.542
eGFR (mL/min/1.73m^2^)	58.9 ± 12.2	68.0 ± 8.4	49.0 ± 6.5	< 0.001
hsCRP (mg/L)	2.9 ± 0.8	2.7 ± 0.7	3.1 ± 0.8	0.034
FGF21 (pg/mL)	350.0 (202.9–820.0)	292.9 (208.3–426.1)	622.2 (200.8–1384.0)	0.093
Medication*s* (*n*, %)				
Insulin	16 (20)	6 (14)	10 (26)	0.267
Sulfonylureas	8 (10)	6 (14)	2 (5)	0.267
Glinides	8 (10)	3 (7)	5 (13)	0.472
GLP-1 RAs	31 (38)	18 (43)	13 (33)	0.493
DPP4 inhibitors	19 (23)	8 (19)	11 (28)	0.433
Metformins	46 (57)	23 (55)	23 (59)	0.823
TZDs	7 (9)	3 (7)	4 (10)	0.706
Alpha-GIs	5 (6)	3 (7)	2 (5)	1.000
SGLT2 inhibitors	37 (46)	17 (40)	20 (51)	0.377
ARBs	65 (80)	36 (86)	29 (74)	0.267
MRAs	17 (21)	8 (19)	9 (23)	0.786
CCBs	22 (27)	11 (26)	11 (28)	1.000
Diuretics	9 (11)	4 (10)	5 (13)	0.732
Statins	64 (79)	36 (86)	28 (72)	0.173
UA lowering agents	20 (25)	10 (24)	10 (26)	1.000

Data are expressed as the mean ± standard deviation, median (interquartile range), or number (%). ALT, alanine transaminase; ARBs, angiotensin receptor blockers; AST, aspartate transaminase; BMI, body mass index; CCBs, calcium channel blockers; DBP, diastolic blood pressure; DKD, diabetic kidney disease; DM, diabetes mellitus; DPP4, dipeptidyl peptidase − 4; eGFR, estimated glomerular filtration rate; FGF21, fibroblast growth factor 21; GIs, glycosidase inhibitors; GLP-1 RAs, glucagon-like peptide − 1 receptor agonists; HbA1c, hemoglobin A1c; HDL-C, high-density lipoprotein cholesterol; hsCRP, high-sensitivity C-reactive protein; LDL-C, low-density lipoprotein cholesterol; MRAs, mineralocorticoid receptor antagonists; SBP, systolic blood pressure; SGLT2, sodium-glucose cotransporter 2; TG, triglycerides; TZDs, thiazolidinediones; T2DM, type 2 diabetes mellitus; UA, uric acid; UACR, urine albumin-to-creatinine ratio

We next examined longitudinal changes in clinical and laboratory parameters after pemafibrate initiation (Table 2). After 2 years of treatment, TG levels decreased significantly from 260 (199–329) to 129 (82–221) mg/dL (*P* < 0.001), whereas HDL-C levels increased from 40 (34–46) to 45 (39–52) mg/dL (*P* < 0.001). AST and ALT levels also decreased significantly during the observation period. Although BMI, blood pressure, HbA1c, LDL-C, and UA did not change significantly, UACR decreased significantly from 27.2 (11.4–41.6) to 15.8 (7.4–27.7) mg/gCr (*P* < 0.001), while eGFR remained stable during the observation period. In addition, hsCRP decreased significantly from 2.9 ± 0.8 to 2.1 ± 1.0 mg/L (*P* < 0.001), whereas circulating FGF21 levels increased from 350.0 (202.9–820.0) to 409.1 (183.0–1200.0) pg/mL (*P* = 0.003). These findings showed significant changes in lipid-related and inflammatory parameters, accompanied by a reduction in albuminuria and an increase in FGF21, while eGFR remained stable during the observation period.

**Table 2:** Changes in parameters between baseline and two years after pemafibrate initiation

	Overall (*n* = 81)		
	Baseline	After	*P-*value
BMI (kg/m^2^)	25.3 ± 3.2	25.2 ± 3.0	0.280
SBP (mmHg)	131 ± 10	131 ± 8	0.902
DBP (mmHg)	70 ± 6	69 ± 6	0.647
HbA1c (%)	8.10 (7.50–8.80)	7.90 (7.30–8.50)	0.225
TG (mg/dL)	260 (199–329)	129 (82–221)	< 0.001
HDL-C (mg/dL)	40 (34–46)	45 (39–52)	< 0.001
LDL-C (mg/dL)	108 (96–117)	111 (92–121)	0.603
AST (U/L)	28 (16–35)	26 (19–31)	< 0.001
ALT (U/L)	39 (29–45)	31 (21–39)	< 0.001
UA (mg/dL)	5.6 (4.1–6.7)	5.6 (3.8–7.3)	0.832
UACR (mg/gCr)	27.2 (11.4–41.6)	15.8 (7.4–27.7)	< 0.001
eGFR (mL/min/1.73m^2^)	58.9 ± 12.2	58.7 ± 12.1	0.867
hsCRP (mg/L)	2.9 ± 0.8	2.1 ± 1.0	< 0.001
FGF21 (pg/mL)	350.0 (202.9–820.0)	409.1 (183.0–1200.0)	0.003

Data are expressed as the mean ± standard deviation, or the median (interquartile range), and are compared using the paired *t*-test or the Wilcoxon signed-rank test. ALT, alanine transaminase; AST, aspartate transaminase; BMI, body mass index; DBP, diastolic blood pressure; eGFR, estimated glomerular filtration rate; FGF21, fibroblast growth factor 21; HbA1c, hemoglobin A1c; HDL-C, high-density lipoprotein cholesterol; hsCRP, high-sensitivity C-reactive protein; LDL-C, low-density lipoprotein cholesterol; SBP, systolic blood pressure; TG, triglycerides; UA, uric acid; UACR, urine albumin-to-creatinine ratio

To determine whether baseline renal status influenced these changes, we performed subgroup analyses according to baseline eGFR and UACR. When patients were stratified by baseline eGFR (Table 3), UACR decreased significantly in both groups, whereas the change in eGFR differed between the groups. In patients with a baseline eGFR of ≥ 60 mL/min/1.73 m^2^, eGFR changed from 68.0 ± 8.4 to 66.2 ± 10.6 mL/min/1.73 m^2^ (*P* = 0.101), with a median change of − 1.6 (− 5.8 to 2.7) mL/min/1.73 m^2^. In contrast, in patients with a baseline eGFR of 30 to < 60 mL/min/1.73 m^2^, eGFR changed from 49.0 ± 6.5 to 50.7 ± 7.6 mL/min/1.73 m^2^ (*P* = 0.089), with a median change of 1.0 (− 3.0 to 5.0) mL/min/1.73 m^2^, which was significantly greater than that in patients with a baseline eGFR of ≥ 60 mL/min/1.73 m^2^. hsCRP decreased significantly in both groups, with a greater reduction in the lower-eGFR group, and FGF21 levels increased significantly in both groups. Consistent with these findings, stratification by baseline UACR showed that the reduction in UACR was significant only in patients with a baseline UACR of ≥ 30 mg/gCr, and the magnitude of the change was greater than that in patients with a baseline UACR of < 30 mg/gCr (Supplementary Table S2). In contrast, changes in eGFR did not differ significantly between the baseline UACR subgroups (Supplementary Table S2).

**Table 3:** Comparison of changes in UACR, eGFR, hsCRP, and FGF21 levels between baseline and two years after pemafibrate initiation based on baseline eGFR

	Baseline	After	*P*-value	Mean change
eGFR ≥ 60 at baseline (*n* = 42)				
UACR (mg/gCr)	26.2 (12.3–37.1)	18.8 (9.7–30.6)	0.017	− 1.5 (− 10.0 to 1.7)
eGFR (mL/min/1.73m^2^)	68.0 ± 8.4	66.2 ± 10.6	0.101	− 1.6 (− 5.8 to 2.7)
hsCRP (mg/L)	2.7 ± 0.7	2.3 ± 1.0	0.008	− 0.7 (− 2.4 to 2.1)
FGF21 (pg/mL)	292.9 (208.3–426.1)	311.1 (179.9–816.4)	0.032	7.3 (− 8.5 to 106.6)
30 ≤ eGFR < 60 at baseline (*n* = 39)				
UACR (mg/gCr)	27.7 (10.4–45.8)	14.4 (7.2–26.7)	0.001	− 5 (− 13.9 to − 1.5)
eGFR (mL/min/1.73m^2^)	49.0 ± 6.5	50.7 ± 7.6	0.089	1.0 (− 3.0 to 5.0) ^*^
hsCRP (mg/L)	3.1 ± 0.8	2.0 ± 0.9	< 0.001	− 1.0 (− 3.1 to 0.4) ^*^
FGF21 (pg/mL)	622.2 (200.8–1384.0)	803 (191.3–1977.8)	0.049	3 (− 4.1 to 186.8)

Data are expressed as the mean ± standard deviation or the median (interquartile range), and were compared using the paired *t*-test or the Wilcoxon signed-rank test. The change in each parameter was calculated by subtracting the baseline value from the value obtained at two years after the initiation of pemafibrate, and data were compared using Student’s *t*-test or the Mann–Whitney *U* test. ^*^P < 0.05 vs. changes in each parameter in patients with an eGFR of ≥ 60 mL/min/1.73 m^2^. CKD, chronic kidney disease; eGFR, estimated glomerular filtration rate; FGF21, fibroblast growth factor 21; hsCRP, high-sensitivity C-reactive protein; UACR, urine albumin-to-creatinine ratio

To further explore factors associated with renal outcomes, we examined correlations between changes in renal parameters and changes in other clinical variables after pemafibrate initiation (Table 4). Spearman’s rank correlation analysis showed that the change in UACR was positively correlated with changes in HbA1c (ρ = 0.234, *P* = 0.036), TG (ρ = 0.559, *P* < 0.001), ALT (ρ = 0.259, *P* = 0.020), hsCRP (ρ = 0.547, *P* < 0.001), and FGF21 (ρ = 0.536, *P* < 0.001). In contrast, the change in eGFR was negatively correlated with changes in TG (ρ =  − 0.302, *P* = 0.006), UACR (ρ =  − 0.400, *P* < 0.001), and FGF21 (ρ =  − 0.519, *P* < 0.001), and showed a borderline negative correlation with the change in hsCRP (ρ =  − 0.217, *P* = 0.052). Thus, changes in FGF21, TG, and hsCRP were associated with changes in renal outcomes during the observation period.

**Table 4:** Correlation analysis of changes in UACR and eGFR with changes in other parameters

	ΔUACR		ΔeGFR	
	*ρ*	*P*-value	*ρ*	*P*-value
ΔBMI (kg/m^2^)	0.045	0.690	− 0.099	0.380
ΔSBP (mmHg)	0.096	0.395	0.050	0.659
ΔDBP (mmHg)	0.019	0.865	− 0.119	0.290
ΔHbA1c (%)	0.234	0.036	− 0.053	0.641
ΔTG (mg/dL)	0.559	< 0.001	− 0.302	0.006
ΔHDL-C (mg/dL)	0.023	0.839	0.117	0.297
ΔLDL-C (mg/dL)	0.107	0.341	− 0.097	0.388
ΔAST (U/L)	0.004	0.969	− 0.023	0.839
ΔALT (U/L)	0.259	0.020	− 0.105	0.350
ΔUA (mg/dL)	− 0.048	0.673	0.120	0.287
ΔUACR (mg/gCr)	N/A	N/A	− 0.400	< 0.001
ΔeGFR (mL/min/1.73m^2^)	− 0.400	< 0.001	N/A	N/A
ΔhsCRP (mg/L)	0.547	< 0.001	− 0.217	0.052
ΔFGF21 (pg/mL)	0.536	< 0.001	− 0.519	< 0.001

Spearman’s rank correlation analysis was performed. The change in each parameter (Δ) was calculated by subtracting the baseline value from the value obtained at two years after the initiation of pemafibrate treatment. ALT, alanine transaminase; AST, aspartate transaminase; BMI, body mass index; DBP, diastolic blood pressure; eGFR, estimated glomerular filtration rate; FGF21, fibroblast growth factor 21; HbA1c, hemoglobin A1c; HDL-C, high-density lipoprotein cholesterol; hsCRP, high-sensitivity C-reactive protein; LDL-C, low-density lipoprotein cholesterol; N/A, not available; SBP, systolic blood pressure; *ρ,* Spearman’s correlation coefficient; TG, triglycerides; UA, uric acid; UACR, urine albumin-to-creatinine ratio

Given the observed associations between FGF21 and renal outcomes in the correlation analyses, we next performed univariate and multivariate linear regression analyses to assess whether these relationships were independent of potential confounders. In the univariate analysis, baseline log-transformed FGF21 levels and the two-year change in log-transformed FGF21 were correlated with the two-year change in eGFR along with three items (Supplementary Table S3). In multivariate models excluding the two-year change in log-transformed FGF21, higher baseline log-transformed FGF21 levels remained associated with a less favorable change in eGFR across all models (Table 5). In multivariate models additionally including the two-year change in log-transformed FGF21, both higher baseline log-transformed FGF21 and a greater increase in log-transformed FGF21 remained associated with a less favorable change in eGFR (Table 5). Similarly, in the univariate analysis, baseline log-transformed FGF21 levels and the two-year change in log-transformed FGF21 were correlated with the two-year change in log-transformed UACR along with four items (Supplementary Table S4). In multivariate models excluding the two-year change in log-transformed FGF21, higher baseline log-transformed FGF21 levels remained associated with a greater increase in log-transformed UACR across all models, and when the change in log-transformed FGF21 was additionally included, both higher baseline log-transformed FGF21 and a greater increase in log-transformed FGF21 remained associated with a greater increase in log-transformed UACR (Supplementary Table S5). These associations were maintained after adjustment for potential confounders, including age, blood pressure, baseline renal parameters, diabetes duration, HbA1c, and TG-related variables. Taken together, these findings indicate that both baseline FGF21 levels and their longitudinal changes remained associated with renal parameter changes in the multivariable models.

**Table 5:** Multivariate linear regression models examining the associations of baseline FGF21 levels and changes in FGF21 with the change in eGFR

Dependent variable: ΔeGFR^†^						
Independent variables	Models excluding ΔLog (FGF21)			Models including ΔLog (FGF21)		
	Unstandardized β	95%CI	*P*-value	Unstandardized β	95%CI	*P*-value
Univariate model						
Log (FGF21)^‡^	− 2.799	− 4.247 to − 1.352	< 0.001	− 2.799	− 4.247 to − 1.352	< 0.001
ΔLog (FGF21)^§^				− 5.789	− 8.280 to − 3.298	< 0.001

Age and gender adjusted model	(Adjusted *R*^2^ = 0.189)			(Adjusted *R*^2^ = 0.305)		
Log (FGF21)^‡^	− 2.280	− 3.760 to − 0.800	0.003	− 1.907	− 3.292 to − 0.522	0.008
ΔLog (FGF21)^§^				− 4.545	− 6.978 to − 2.111	< 0.001
Age	− 0.170	− 0.314 to − 0.027	0.020	− 0.129	− 0.263 to 0.006	0.061
Female sex	− 0.549	− 3.282 to 2.185	0.690	− 0.041	− 2.587 to 2.505	0.975

Liver enzymes adjusted model	(Adjusted *R*^2^ = 0.133)			(Adjusted *R*^2^ = 0.282)		
Log (FGF21)^‡^	− 2.808	− 4.272 to − 1.344	< 0.001	− 2.221	− 3.583 to − 0.858	0.002
ΔLog (FGF21)^§^				− 5.013	− 7.434 to − 2.592	< 0.001
Log (AST)^‡^	− 0.454	− 4.615 to 3.707	0.828	− 0.496	− 4.283 to 3.291	0.795
Log (ALT)^‡^	− 1.312	− 6.574 to 3.949	0.621	− 1.575	− 6.366 to 3.215	0.514

Multivariate model 1	(Adjusted *R*^2^ = 0.384)			(Adjusted *R*^2^ = 0.464)		
Log (FGF21)^‡^	− 3.629	− 5.052 to − 2.296	< 0.001	− 3.177	− 4.530 to − 1.824	< 0.001
ΔLog (FGF21)^§^				− 3.788	− 5.937 to − 1.639	< 0.001
Age	− 0.089	− 0.219 to 0.040	0.172	− 0.060	− 0.181 to 0.062	0.334
SBP	− 0.109	− 0.232 to 0.014	0.081	− 0.093	− 0.208 to 0.022	0.110
eGFR	− 0.263	− 0.372 to − 0.155	< 0.001	− 0.240	− 0.342 to − 0.138	< 0.001

Multivariate model 2	(Adjusted *R*^2^ = 0.373)			(Adjusted *R*^2^ = 0.448)		
Log (FGF21)^‡^	− 3.602	− 5.112 to − 2.092	< 0.001	− 3.054	− 4.509 to − 1.598	< 0.001
ΔLog (FGF21)^§^				− 3.758	− 6.033 to − 1.483	0.002
Age	− 0.079	− 0.212 to 0.055	0.245	− 0.050	− 0.176 to 0.077	0.438
SBP	− 0.111	− 0.238 to 0.016	0.085	-0.102	-0.222 to 0.017	0.092
eGFR	− 0.262	− 0.372 to − 0.152	< 0.001	− 0.241	− 0.344 to − 0.137	< 0.001
Log (UACR)^‡^	0.308	− 0.807 to 1.423	0.584	− 0.099	− 1.175 to 0.976	0.854
Log (Duration of DM)^‡^	− 0.744	− 1.990 to 0.502	0.238	− 0.481	− 1.662 to 0.700	0.420
Log (HbA1c)^‡^	1.274	− 9.348 to 11.895	0.812	− 0.323	− 10.343 to 9.696	0.949

Multivariate model 3	(Adjusted *R*^2^ = 0.378)			(Adjusted *R*^2^ = 0.462)		
Log (FGF21)^‡^	− 3.614	− 5.117 to − 2.112	< 0.001	− 2.946	− 4.416 to − 1.477	< 0.001
ΔLog (FGF21)^§^				− 4.190	− 6.498 to − 1.883	< 0.001
Age	− 0.099	− 0.231 to 0.032	0.137	− 0.066	− 0.191 to 0.060	0.300
SBP	− 0.102	− 0.228 to 0.024	0.110	− 0.092	− 0.210 to 0.026	0.126
eGFR	− 0.257	− 0.366 to − 0.147	< 0.001	− 0.234	− 0.339 to − 0.129	< 0.001
Log (UACR)^‡^	0.057	− 1.065 to 1.178	0.920	− 0.434	− 1.517 to 0.650	0.427
Log (TG)^‡^	2.316	− 2.104 to 6.736	0.300	3.383	− 0.801 to 7.568	0.111
ΔLog (TG)^§¶^				0.337	− 2.165 to 2.840	0.789

A multivariate linear regression analysis with crude and adjusted beta coefficients. All independent variables, except for changes in parameters, corresponded to the values obtained at baseline. ALT, alanine transaminase; AST, aspartate transaminase; CI, confidence interval; DM, diabetes mellitus; eGFR, estimated glomerular filtration rate; FGF21, fibroblast growth factor 21; HbA1c, hemoglobin A1c; β, regression coefficient; SBP, systolic blood pressure; TG, triglycerides; UACR, urine albumin-to-creatinine ratio. ^†^Change in eGFR (mL/min/1.73 m^2^) from baseline to two years after pemafibrate initiation. ^‡^Log-transformed using the natural logarithm. ^§^Changes in log-transformed parameters, calculated using the natural logarithm, from baseline to two years after pemafibrate initiation. ^¶^Entered only into the models including changes in log-transformed FGF21 levels

## Discussion

In this retrospective observational study of patients with T2DM, triglyceride, liver enzyme, hsCRP, and UACR levels decreased during the two-year observation period after pemafibrate initiation, whereas eGFR remained stable overall. In the subgroup analysis, the two-year change in eGFR was significantly more favorable in patients with a baseline eGFR of 30 to < 60 mL/min/1.73 m^2^ than in those with a baseline eGFR of ≥ 60 mL/min/1.73 m^2^. Furthermore, higher baseline FGF21 levels and greater increases in FGF21 remained associated with less favorable changes in eGFR and UACR in multivariable analyses. These findings demonstrate an association between circulating FGF21 status and renal parameter changes in this cohort but do not establish a causal role of FGF21 or a specific effect of pemafibrate.

Recent evidence indicates that dysregulated lipid metabolism and impaired fatty acid oxidation contribute to DKD progression [25, 26]. PPARα regulates fatty acid β-oxidation, mitochondrial energy metabolism, and inflammatory responses [27]. Moreover, PPARα has been reported to directly suppress several inflammatory signaling pathways, including NOD-like receptor protein 3 inflammasome [28], nuclear factor-kappa B [29], transforming growth factor beta [30], and activator protein 1 [30]. Experimental studies have shown that PPARα deficiency exacerbates diabetic kidney injury, whereas pharmacological activation of PPARα by fenofibrate, a conventional PPARα agonist, restores fatty acid oxidation and inflammatory responses, leading to improvements in albuminuria, renal inflammation, and fibrosis in diabetic models [7, 31, 32].

Pemafibrate has attracted attention as a selective PPARα modulator that can be used in patients with impaired renal function. Experimental studies have demonstrated effects of pemafibrate on oxidative stress, fatty acid metabolism, and inflammatory pathways in models of kidney disease [13, 14, 33]. In the present cohort, hsCRP and UACR decreased in parallel during the observation period. Although these observations are compatible with the biological pathways demonstrated experimentally, the absence of a control group prevents attribution of these longitudinal changes specifically to pemafibrate or determination of the mechanisms underlying them.

In the overall cohort, eGFR remained stable during the two-year observation period. Previous observational studies have also reported relatively stable renal function during pemafibrate treatment [12, 34]. Interestingly, the change in eGFR was more favorable in patients with a baseline eGFR of 30 to < 60 mL/min/1.73 m^2^ than in those with an eGFR of ≥ 60 mL/min/1.73 m^2^. Several experimental studies suggest that abnormalities in PPARα-related fatty acid metabolism and inflammatory pathways become more prominent with kidney dysfunction [14, 26, 27], providing a possible biological context for differential associations according to baseline renal function. However, because the subgroups were defined according to baseline eGFR, regression to the mean may have contributed, at least in part, to the observed between-group difference in eGFR change. In addition, the absolute magnitude of the between-group difference in eGFR change was small. Given the biological and analytical variability inherent in eGFR measurements, the statistical significance of this finding should not be assumed to indicate clinical significance. Moreover, because the present study lacked a control group and renal function was assessed at only two time points, this subgroup finding should be interpreted cautiously and cannot establish a differential treatment effect of pemafibrate according to baseline eGFR.

The relationship between circulating FGF21 and renal parameter changes requires careful interpretation. In the present study, higher baseline FGF21 levels and greater increases in FGF21 after pemafibrate initiation were associated with less favorable changes in eGFR and UACR. Both fenofibrate and pemafibrate are known to increase circulating FGF21 through PPARα activation in humans [17, 35], and experimental studies have suggested that FGF21 participates in pathways related to renoprotection, including attenuation of oxidative stress and inflammatory signaling [15, 16]. However, these experimental biological effects should not be interpreted as indicating that higher circulating FGF21 concentrations necessarily represent a favorable response in clinical populations. In contrast to experimental findings regarding the biological actions of FGF21, observational studies in patients with T2DM have reported that elevated circulating FGF21 levels are associated with albuminuria and subsequent kidney function decline [18, 19]. Elevated endogenous FGF21 concentrations are also commonly observed in obesity and T2DM despite the favorable metabolic effects of FGF21 demonstrated experimentally. This apparent discrepancy has led to the proposed concept of FGF21 resistance, in which increased circulating FGF21 may coexist with impaired receptor expression or downstream signaling [20, 21]. In addition, elevated FGF21 may represent a compensatory response to systemic metabolic stress rather than directly contributing to disease progression. Another important possibility is confounding by underlying disease severity. Baseline renal dysfunction or systemic metabolic stress may independently lead to higher circulating FGF21 concentrations while also predisposing patients to subsequent deterioration in renal parameters. Thus, the observed association between FGF21 and renal parameter changes may arise, at least in part, from a common underlying pathophysiological state rather than from a direct effect of FGF21 itself. Moreover, because changes in FGF21 and renal parameters were assessed over the same two-year interval, their temporal sequence cannot be established, and reverse causation cannot be excluded. Accordingly, our findings should not be interpreted as demonstrating that an increase in FGF21 causes renal deterioration or, conversely, that a smaller FGF21 increase mediates the renal effects of pemafibrate. The observed association may indicate that higher or increasing circulating FGF21 reflects greater underlying metabolic or renal stress, a compensatory endocrine response, impaired FGF21 responsiveness, or a combination of these processes. Because FGF21 receptor expression and downstream signaling were not evaluated in the present study, FGF21 resistance remains a possible but unproven explanation. Further mechanistic and prospective clinical studies are required to clarify the biological basis of the association between FGF21 dynamics and renal outcomes. From a clinical perspective, the observed associations of baseline and longitudinal FGF21 levels with renal parameter changes suggest that circulating FGF21 may warrant further investigation as a marker of renal risk during follow-up. However, the present study did not establish predictive performance or a clinically meaningful cutoff value, and no independent validation cohort was available. Therefore, these findings should be considered hypothesis-generating rather than evidence supporting the current clinical use of FGF21 as a prognostic biomarker.

Several limitations should be considered. First, the relatively small sample size limited the number of covariates that could be reliably included in the multivariable models. In addition, because a relatively large number of candidate variables were considered using a stepwise variable-selection procedure, model instability and overfitting cannot be excluded. Therefore, the associations observed in the multivariable analyses should be interpreted cautiously and require confirmation in larger, independent cohorts. Second, multiple statistical comparisons were performed in exploratory subgroup, correlation, and multivariable analyses, without a prespecified adjustment for multiplicity. Therefore, the possibility of type I error due to multiple testing cannot be excluded, and individual P-values should be interpreted as exploratory and hypothesis-generating rather than confirmatory. Third, because this was a retrospective observational study without an untreated or otherwise appropriate control group, causal relationships cannot be inferred. The observed longitudinal changes cannot be attributed specifically to pemafibrate and may have been influenced by background clinical care, changes in lifestyle or concomitant treatment, temporal changes in metabolic control, the natural course of diabetes and kidney disease, and residual confounding.

Fourth, 65 of the 146 potentially eligible patients were excluded from the analysis, including patients with insufficient stored serum samples. Although the available baseline characteristics were generally comparable between included and excluded patients, with no statistically significant differences observed in the evaluated parameters, the availability of residual serum samples may not have occurred completely at random. Therefore, selection bias related to unmeasured characteristics, the frequency or timing of clinical visits and sample collection, or sample availability cannot be excluded and may limit the generalizability of the findings. Fifth, regression to the mean should be considered when interpreting both the reduction in UACR and the between-group difference in eGFR change because the subgroup analyses were defined according to baseline renal parameters. In addition, although the between-group difference in eGFR change reached statistical significance, its absolute magnitude was small and should be interpreted in the context of the biological and analytical variability of eGFR measurements; therefore, its clinical relevance remains uncertain. UACR is also subject to substantial biological and analytical variability, and the use of spot urine measurements at only two time points may not fully capture the longitudinal burden of albuminuria. Sixth, renal parameters and FGF21 levels were evaluated only at baseline and two years after pemafibrate initiation, precluding assessment of their temporal trajectories and the temporal sequence of changes. Therefore, reverse causation cannot be excluded. Moreover, baseline renal dysfunction or systemic metabolic stress may have independently contributed to both elevated circulating FGF21 levels and subsequent unfavorable changes in renal parameters, resulting in residual confounding by underlying disease severity. In addition, changes in medications other than SGLT2 inhibitors, GLP-1RAs, ARBs, and MRAs were not fully controlled during follow-up. The two-year observation period may also have been insufficient to evaluate clinically meaningful long-term changes in kidney function. Finally, the very limited use of pemafibrate in patients with an eGFR of < 30 mL/min/1.73 m^2^ at our institution precluded evaluation of this population, thereby limiting the generalizability of the findings to patients with advanced CKD.

In conclusion, higher baseline FGF21 levels and greater increases in FGF21 remained associated with less favorable changes in renal parameters in multivariable analyses during the two-year observation period after pemafibrate initiation in patients with T2DM. These associations do not establish a causal role of FGF21 or a specific renal effect of pemafibrate. The present findings should therefore be considered hypothesis-generating and provide a rationale for prospective controlled studies to determine the clinical significance and predictive utility of circulating FGF21 in patients with T2DM and DKD.

## Supplementary Information

Below is the link to the electronic supplementary material.

Supplementary file1 (DOCX 62 KB)

## Data Availability

The datasets analyzed during the current study are not publicly available because they contain potentially identifiable clinical information, but are available from the corresponding author on reasonable request and with appropriate institutional approval.

## References

[1] Alicic RZ, Rooney MT, Tuttle KR. Diabetic kidney disease: challenges, progress, and possibilities. Clin J Am Soc Nephrol. 2017;12(12):2032–45. 10.2215/CJN.11491116 PMC5718284 28522654

[2] Hang X, Ma J, Wei Y, Wang Y, Zang X, Xie P, et al. Renal microcirculation and mechanisms in diabetic kidney disease. Front Endocrinol (Lausanne). 2025;16:1580608. 10.3389/fendo.2025.1580608 PMC12176566 40538805

[3] Pugliese G, Penno G, Natali A, Barutta F, Di Paolo S, Reboldi G, et al. Diabetic kidney disease: new clinical and therapeutic issues. Joint position statement of the Italian Diabetes Society and the Italian Society of Nephrology on “The natural history of diabetic kidney disease and treatment of hyperglycemia in patients with type 2 diabetes and impaired renal function.” J Nephrol. 2020;33(1):9–35. 10.1007/s40620-019-00650-x PMC7007429 31576500

[4] Naaman SC, Bakris GL. Diabetic nephropathy: update on pillars of therapy slowing progression. Diabetes Care. 2023;46(9):1574–86. 10.2337/dci23-0030 PMC10547606 37625003

[5] Yang S, Zhao L, Mi Y, He W. Effects of sodium-glucose cotransporter-2 inhibitors and aldosterone antagonists, in addition to renin-angiotensin system antagonists, on major adverse kidney outcomes in patients with type 2 diabetes and chronic kidney disease: a systematic review and network meta-analysis. Diabetes Obes Metab. 2022;24(11):2159–68. 10.1111/dom.14801 35712807

[6] Aomura D, Nimura T, Yamaka K, Yamada Y, Harada M, Nakajima T, et al. Proximal tubule-specific genetic deficiency of PPARα worsens systemic lipid and glucose metabolism during fasting. FASEB J. 2026;40(1):e71333. 10.1096/fj.202502975R PMC12747219 41460648

[7] Tanaka Y, Kume S, Araki S, Isshiki K, Chin-Kanasaki M, Sakaguchi M, et al. Fenofibrate, a PPARα agonist, has renoprotective effects in mice by enhancing renal lipolysis. Kidney Int. 2011;79(8):871–82. 10.1038/ki.2010.530 21270762

[8] Japanese Society of Nephrology. Essential points from evidence-based clinical practice guideline for chronic kidney disease 2023. Clin Exp Nephrol. 2024;28(6):473–95. 10.1007/s10157-024-02497-4 PMC11116248 38713253

[9] Fruchart JC. Pemafibrate (K-877), a novel selective peroxisome proliferator-activated receptor alpha modulator for management of atherogenic dyslipidaemia. Cardiovasc Diabetol. 2017;16(1):124. 10.1186/s12933-017-0602-y PMC5628452 28978316

[10] Yamashita S. Clinical applications of a novel selective PPARα modulator, pemafibrate, in dyslipidemia and metabolic diseases. J Atheroscler Thromb. 2019;26(5):389–402. 10.5551/jat.48918 PMC6514171 30930344

[11] Ishibashi S, Arai H, Yokote K, Araki E, Suganami H, Yamashita S. Pharmacokinetics and safety of pemafibrate in patients with both dyslipidemia and severe renal impairment: a phase 4 study. J Atheroscler Thromb. 2025;32(2):210–25. 10.5551/jat.64887 PMC11802250 39231654

[12] Izumihara R, Nomoto H, Kito K, Yamauchi Y, Omori K, Shibayama Y, et al. Switching from conventional fibrates to pemafibrate has beneficial effects on the renal function of diabetic subjects with chronic kidney disease. Diabetes Metab J. 2024;48(3):473–81. 10.4093/dmj.2023.0370 PMC11140405 38419467

[13] Horinouchi Y, Murashima Y, Yamada Y, Aomura D, Nishiyama T, Shimizu M, et al. Pemafibrate inhibited renal dysfunction and fibrosis in a mouse model of adenine-induced chronic kidney disease. Life Sci. 2023;15(321):121590. 10.1016/j.lfs.2023.121590 36940907

[14] Aomura D, Harada M, Yamada Y, Nakajima T, Hashimoto K, Kanno H, et al. Pemafibrate protects against fatty acid-induced nephropathy by maintaining renal fatty acid metabolism. Metabolites. 2021;11(6):372. 10.3390/metabo11060372 PMC8230306 34207854

[15] Cheng Y, Zhang J, Guo W, Li F, Sun W, Chen J, et al. Up-regulation of Nrf2 is involved in FGF21-mediated fenofibrate protection against type 1 diabetic nephropathy. Free Radic Biol Med. 2016;93:94–109. 10.1016/j.freeradbiomed.2016.02.002 PMC7446394 26849944

[16] Takahara K, Ouchi N, Takikawa T, Ozaki Y, Fang L, Kawanishi H, et al. Pemafibrate ameliorates renal injury through induction of FGF21 and ketone body production in male mice. Physiol Rep. 2025;13(3):e70135. 10.14814/phy2.70135 PMC11780494 39887648

[17] Ong KL, O’Connell R, Januszewski AS, Jenkins AJ, Xu A, Sullivan DR, et al. Baseline circulating FGF21 concentrations and increase after fenofibrate treatment predict more rapid glycemic progression in type 2 diabetes: a FIELD substudy. Clin Chem. 2017;63(7):1261–70. 10.1373/clinchem.2016.270876 28606915

[18] Lee CH, Hui EYL, Woo YC, Yeung CY, Chow WS, Yuen MMA, et al. Circulating fibroblast growth factor 21 levels predict progressive kidney disease in subjects with type 2 diabetes and normoalbuminuria. J Clin Endocrinol Metab. 2015;100(4):1368–75. 10.1210/jc.2014-3465 25625802

[19] de Albuquerque Suassuna PG, de Paula RB, Sanders-Pinheiro H, Moe OW, Hu MC. Fibroblast growth factor 21 in chronic kidney disease. J Nephrol. 2019;32(3):365–77. 10.1007/s40620-018-0550-y PMC6483847 30430412

[20] Fisher FM, Chui PC, Antonellis PJ, Bina HA, Kharitonenkov A, Flier JS, et al. Obesity is a fibroblast growth factor 21 (FGF21)-resistant state. Diabetes. 2010;59(11):2781–9. 10.2337/db10-0193 PMC2963536 20682689

[21] Gan J, Duan Z, Tang L, Wu M, Wang X, Luo Y, et al. Fibroblast growth factor 21 resistance is associated with body shape in patients with type 2 diabetes complicating hypertension. Front Cardiovasc Med. 2023;22(10):1168047. 10.3389/fcvm.2023.1168047 PMC10325616 37424900

[22] Levey AS, Gassman JJ, Hall PM, Walker WG. Assessing the progression of renal disease in clinical studies: effects of duration of follow-up and regression to the mean. Modification of Diet in Renal Disease (MDRD) Study Group. J Am Soc Nephrol. 1991;1(9):1087–94. 10.1681/ASN.V191087 1912407

[23] Khwaja A. KDIGO clinical practice guidelines for acute kidney injury. Nephron Clin Pract. 2012;120(4):c179–84. 10.1159/000339789 22890468

[24] Horio M, Imai E, Yasuda Y, Watanabe T, Matsuo S. Modification of the CKD epidemiology collaboration equation for Japanese: accuracy and use for population estimates. Am J Kidney Dis. 2010;56(1):32–8. 10.1053/j.ajkd.2010.02.344 20416999

[25] Han YZ, Du BX, Zhu XY, Jiang L, Xiong Y, Zhou XJ, et al. Lipid metabolism disorder in diabetic kidney disease. Front Endocrinol (Lausanne). 2024;29(15):1336402. 10.3389/fendo.2024.1336402 PMC11089115 38742197

[26] Kang HM, Ahn SH, Choi P, Ko YA, Han SH, Chinga F, et al. Defective fatty acid oxidation in renal tubular epithelial cells has a key role in kidney fibrosis development. Nat Med. 2015;21(1):37–46. 10.1038/nm.3762 PMC4444078 25419705

[27] Li S, Nagothu KK, Desai V, Lee T, Branham W, Moland C, et al. Transgenic expression of proximal tubule peroxisome proliferator-activated receptor-alpha in mice confers protection during acute kidney injury. Kidney Int. 2009;76(10):1049–62. 10.1038/ki.2009.330 PMC3244273 19710628

[28] Grabacka M, Pierzchalska M, Płonka PM, Pierzchalski P. The role of PPAR alpha in the modulation of innate immunity. Int J Mol Sci. 2021;22(19):10545. 10.3390/ijms221910545 PMC8508635 34638886

[29] Decara J, Rivera P, López-Gambero AJ, Serrano A, Pavón FJ, Baixeras E, et al. Peroxisome proliferator-activated receptors: experimental targeting for the treatment of inflammatory bowel diseases. Front Pharmacol. 2020;11:730. 10.3389/fphar.2020.00730 PMC7266982 32536865

[30] Masenga SK, Desta S, Hatcher M, Harris SP. How PPAR-alpha mediated inflammation may affect the pathophysiology of chronic kidney disease. Curr Res Physiol. 2024;14(8):100133. 10.1016/j.crphys.2024.100133 PMC11629568 39665027

[31] Park CW, Zhang Y, Zhang X, Wu J, Chen L, Cha DR, et al. Accelerated diabetic nephropathy in mice lacking the peroxisome proliferator-activated receptor alpha. Diabetes. 2006;55(4):885–93. 10.2337/diabetes.55.04.06.db05-1329 16567507

[32] Chen LL, Zhang JY, Wang BP. Renoprotective effects of fenofibrate in diabetic rats are achieved by suppressing kidney plasminogen activator inhibitor-1. Vascul Pharmacol. 2006;44(5):309–15. 10.1016/j.vph.2006.01.004 16624630

[33] Maki T, Maeda Y, Sonoda N, Makimura H, Kimura S, Maeno S, et al. Renoprotective effect of a novel selective PPARα modulator K-877 in db/db mice: a role of diacylglycerol-protein kinase C-NAD(P)H oxidase pathway. Metabolism. 2017;71:33–45. 10.1016/j.metabol.2017.02.013 28521876

[34] Iwasaki M, Suzuki H, Umezawa Y, Hasegawa K, Okada K, Wakino S, et al. Efficacy and safety of pemafibrate in patients with chronic kidney disease: a retrospective study. Medicine (Baltimore). 2023;102(7):e32818. 10.1097/MD.0000000000032818 PMC9936022 36800602

[35] Gälman C, Lundåsen T, Kharitonenkov A, Bina HA, Eriksson M, Hafström I, et al. The circulating metabolic regulator FGF21 is induced by prolonged fasting and PPARalpha activation in man. Cell Metab. 2008;8(2):169–74. 10.1016/j.cmet.2008.06.014 18680716

