# Supplementary material for: Associations of fibroblast growth factor 21 with renal parameter changes during pemafibrate treatment in patients with type 2 diabetes
Source: Diabetol Int. 2026 Nov 10;18(1):6. doi: 10.1007/s13340-026-00945-y (PMC13748181; doi:10.1007/s13340-026-00945-y)
Supplement: Supplementary file 1 [file 13340_2026_945_MOESM1_ESM.docx]

**SUPPLEMENTARY TABLE**

**Supplementary Table S1. Comparison of baseline characteristics between included and excluded participants**

|  | Included participants  (*n* = 81) | Excluded participants  (*n* = 65) | *P-*value |
| --- | --- | --- | --- |
| Clinical characteristics |  |  |  |
| Age (years) | 56.8±9.8 | 57.6±13.1 | 0.667 |
| Female sex, *n* (%) | 41 (51) | 27 (42) | 0.318 |
| Duration of DM (years) | 11 (5-19) | 10 (2-18) | 0.186 |
| Current Smoker (*n*, %) | 26 (32) | 16 (25) | 0.361 |
| BMI (kg/m^2^) | 25.3±3.2 | 26.0±4.8 | 0.282 |
| Complications of DM (*n*, %) |  |  |  |
| Neuropathy | 42 (52) | 40 (62) | 0.314 |
| Retinopathy | 30 (37) | 26 (40) | 0.735 |
| DKD | 57 (70) | 43 (66) | 0.596 |
| Atherosclerotic diseases | 20 (25) | 20 (31) | 0.458 |
| Classification based on eGFR (mL/min/1.73m^2^) or UACR (mg/gCr) |  |  |  |
| eGFR ≥90 | 4 (5) | 1 (2) | 0.504 |
| 60 ≤ eGFR < 90 | 38 (47) | 26 (40) |  |
| 30 ≤ eGFR < 60 | 39 (48) | 27 (42) |  |
| eGFR <30 | 0 (0) | 0 (0) |  |
| UACR <30 | 44 (54) | 37 (57) | 0.944 |
| 30 ≤ UACR < 300 | 35 (43) | 27 (42) |  |
| UACR ≥300 | 2 (3) | 1 (2) |  |
| Physiological testing |  |  |  |
| SBP (mmHg) | 131±10 | 133±17 | 0.356 |
| DBP (mmHg) | 70±6 | 71±8 | 0.341 |
| Laboratory parameters |  |  |  |
| HbA1c (%) | 8.10 (7.50–8.80) | 8.50 (7.60–9.10) | 0.101 |
| TG (mg/dL) | 260 (199–329) | 280 (204–349) | 0.432 |
| HDL-C (mg/dL) | 40 (34–46) | 41 (36–46) | 0.445 |
| LDL-C (mg/dL) | 108 (96–117) | 111 (101–123) | 0.108 |
| AST (U/L) | 28 (16–35) | 30 (22–38) | 0.182 |
| ALT (U/L) | 39 (29–45) | 37 (27–46) | 0.786 |
| UA (mg/dL) | 5.6 (4.1–6.7) | 5.3 (3.9–6.4) | 0.397 |
| UACR (mg/gCr) | 27.2 (11.4–41.6) | 23.6 (9.4–39.6) | 0.522 |
| eGFR (mL/min/1.73m^2^) | 58.9±12.2 | 61.7±13.4 | 0.187 |
| Medications (*n*, %) |  |  |  |
| Insulin | 16 (20) | 11 (17) | 0.831 |
| Sulfonylureas | 8 (10) | 11 (17) | 0.226 |
| Glinides | 8 (10) | 10 (15) | 0.325 |
| GLP-1 RAs | 31 (38) | 23 (35) | 0.734 |
| DPP4 inhibitors | 19 (23) | 14 (22) | 0.844 |
| Metformins | 46 (57) | 38 (58) | 0.868 |
| TZDs | 7 (9) | 10 (15) | 0.299 |
| Alpha-GIs | 5 (6) | 6 (9) | 0.540 |
| SGLT2 inhibitors | 37 (46) | 23 (35) | 0.238 |
| ARBs | 65 (80) | 54 (83) | 0.831 |
| MRAs | 17 (21) | 19 (29) | 0.334 |
| CCBs | 22 (27) | 12 (18) | 0.242 |
| Diuretics | 9 (11) | 2 (3) | 0.112 |
| Statins | 64 (79) | 54 (83) | 0.673 |
| UA lowering agents | 20 (25) | 19 (29) | 0.576 |

Data are expressed as the mean ± standard deviation, median (interquartile range), or number (%). Continuous variables were compared using the t-test or Mann–Whitney U test, and categorical variables were compared using the Chi-square test or Fisher’s exact test, as appropriate. Available baseline data were used for each comparison. ALT, alanine transaminase; ARBs, angiotensin receptor blockers; AST, aspartate transaminase; BMI, body mass index; CCBs, calcium channel blockers; DBP, diastolic blood pressure; DKD, diabetic kidney disease; DM, diabetes mellitus; DPP4, dipeptidyl peptidase-4; eGFR, estimated glomerular filtration rate; GIs, glycosidase inhibitors; GLP-1 RAs, glucagon-like peptide-1 receptor agonists; HbA1c, hemoglobin A1c; HDL-C, high-density lipoprotein cholesterol; LDL-C, low-density lipoprotein cholesterol; MRAs, mineralocorticoid receptor antagonists; SBP, systolic blood pressure; SGLT2, sodium-glucose cotransporter 2; TG, triglycerides; TZDs, thiazolidinediones; T2DM, type 2 diabetes mellitus; UA, uric acid; UACR, urine albumin-to-creatinine ratio.

**Supplementary Table S2. Comparison of changes in UACR, eGFR, hsCRP, and FGF21 levels between baseline and two years after pemafibrate initiation based on baseline UACR**

|  | Baseline | After | *P*-value | Mean change |
| --- | --- | --- | --- | --- |
| **UACR <30**  **at baseline (*n* = 44)** |  | | | |
| UACR (mg/gCr) | 11.4 (7.3–19.1) | 11.9 (5.4–18.6) | 0.444 | -1.2 (-4.4 to 4.2) |
| eGFR (mL/min/1.73m^2^) | 60.4±11.4 | 60.5±11.7 | 0.951 | -0.8 (-3.9 to 4.0) |
| hsCRP (mg/L) | 2.8±0.8 | 2.2±1.0 | <0.001 | -0.5 (-1.0 to -0.1) |
| FGF21 (pg/mL) | 272.0 (204.1–622.2) | 305.6 (181.8–941.6) | 0.006 | 6.2 (-3.0 to 187.9) |
| **UACR ≥30**  **at baseline (*n* = 37)** |  | | | |
| UACR (mg/gCr) | 43.1 (37.1–163.4) | 26.7 (15.3–49.5) | <0.001 | -12.9 (-24.7 to -4.5) ^***^ |
| eGFR (mL/min/1.73m^2^) | 57.1±12.9 | 56.7±12.4 | 0.721 | 0 (-3.6 to 3.0) |
| hsCRP (mg/L) | 3.1±0.8 | 2.1±0.9 | <0.001 | -1.1 (-1.7 to -0.5) ^*^ |
| FGF21 (pg/mL) | 411.8 (202.9–1350.0) | 507.4 (184.0–1451.6) | 0.213 | 5.0 (-14.1 to 101.0) |

Data are expressed as the mean ± standard deviation or the median (interquartile range), and were compared using the paired *t*-test or the Wilcoxon signed-rank test. The change in each parameter was calculated by subtracting the baseline value from the value obtained at two years after the initiation of pemafibrate, and data were compared using Student’s *t*-test or the Mann–Whitney *U* test. ^*^*P* < 0.05, ^***^*P* < 0.001 vs. the change in each parameter in patients with a UACR of <30 mg/gCr. eGFR, estimated glomerular filtration rate; FGF21, fibroblast growth factor 21; hsCRP, high-sensitivity C-reactive protein; UACR, urine albumin-to-creatinine ratio.

**Supplementary Table S3. Univariate linear regression models examining the association between two-year change in eGFR and each clinical parameter**

| Dependent variables: ΔeGFR ^†^ | | | |
| --- | --- | --- | --- |
| Independent variables | Unstandardized β | 95%CI | *P*-value |
| Clinical characteristics |  |  |  |
| Age | -0.234 | -0.378 to -0.090 | 0.002 |
| Female sex | -1.837 | -4.793 to 1.120 | 0.220 |
| Log (Duration of DM) ^‡^ | -1.341 | -2.793 to 0.111 | 0.070 |
| Current Smoker | 1.022 | -2.166 to 4.210 | 0.525 |
| BMI | -0.187 | -0.657 to 0.282 | 0.430 |
| Complications of DM |  |  |  |
| Neuropathy | -0.127 | -3.113 to 2.860 | 0.933 |
| Retinopathy | 0.087 | -3.003 to 3.177 | 0.956 |
| Atherosclerotic diseases | -2.882 | -6.282 to 0.518 | 0.096 |
| Physiological testing |  |  |  |
| SBP | -0.121 | -0.274 to 0.032 | 0.118 |
| DBP | 0.126 | -0.120 to 0.372 | 0.312 |
| Laboratory parameters |  |  |  |
| Log (HbA1c) ^‡^ | 4.771 | -8.334 to 17.875 | 0.471 |
| Log (TG) ^‡^ | 2.883 | -2.414 to 8.180 | 0.282 |
| ΔLog (TG) ^§^ | -3.602 | -6.500 to -0.704 | 0.016 |
| Log (HDL-C) ^‡^ | -1.874 | -11.111 to 7.363 | 0.687 |
| Log (LDL-C) ^‡^ | 1.360 | -10.154 to 12.873 | 0.815 |
| Log (AST) ^‡^ | -1.305 | -4.813 to 2.203 | 0.461 |
| Log (ALT) ^‡^ | -1.404 | -5.844 to 3.036 | 0.531 |
| Log (UA) ^‡^ | 1.815 | -2.611 to 6.241 | 0.417 |
| Log (UACR) ^‡^ | -0.449 | -1.703 to 0.804 | 0.478 |
| eGFR | -0.159 | -0.277 to -0.041 | 0.009 |
| hsCRP | -0.437 | -2.349 to 1.475 | 0.650 |
| Log (FGF21) ^‡^ | -2.799 | -4.247 to -1.352 | <0.001 |
| ΔLog (FGF21) ^§^ | -5.789 | -8.280 to -3.298 | <0.001 |
| Medications |  |  |  |
| Insulin | 0.786 | -2.958 to 4.530 | 0.677 |
| Sulfonylureas | 1.082 | -3.914 to 6.077 | 0.668 |
| Glinides | -1.304 | -6.297 to 3.689 | 0.605 |
| GLP-1 RAs | 1.383 | -1.671 to 4.437 | 0.370 |
| DPP4 inhibitors | -0.985 | -4.500 to 2.529 | 0.578 |
| Metformins | 1.717 | -1.270 to 4.705 | 0.256 |
| TZDs | 4.687 | -0.519 to 9.893 | 0.077 |
| Alpha-GIs | -3.256 | -9.414 to 2.901 | 0.296 |
| SGLT2 inhibitors | -1.014 | -4.001 to 1.972 | 0.501 |
| ARBs | -1.861 | -5.586 to 1.864 | 0.323 |
| MRAs | -0.058 | -3.723 to 3.606 | 0.975 |
| CCBs | -2.787 | -6.083 to 0.510 | 0.096 |
| Diuretics | -1.435 | -6.172 to 3.303 | 0.548 |
| Statins | 0.572 | -3.090 to 4.234 | 0.757 |
| UA lowering agents | -0.724 | -4.181 to 2.733 | 0.678 |

All independent variables, except for changes in parameters, corresponded to the values obtained at baseline. ALT, alanine transaminase; ARBs, angiotensin receptor blockers; AST, aspartate transaminase; BMI, body mass index; CCB, calcium channel blocker; CI, confidence interval; DBP, diastolic blood pressure; DM, diabetes mellitus; DPP4, dipeptidyl peptidase-4; eGFR, estimated glomerular filtration rate; FGF21, fibroblast growth factor 21; GIs, glycosidase inhibitors; GLP-1 RA, glucagon-like peptide-1 receptors agonist; HbA1c, hemoglobin A1c; HDL-C, high-density lipoprotein cholesterol; hsCRP, high-sensitivity C-reactive protein; LDL-C, low-density lipoprotein cholesterol; MRAs, mineralocorticoid receptor antagonists; β, regression coefficient; SBP, systolic blood pressure; SGLT2, sodium-glucose cotransporter 2; TG, triglycerides; TZDs, thiazolidinediones; T2DM, type 2 diabetes mellitus; UA, uric acid; UACR, urine albumin-to-creatinine ratio. ^†^ Change in eGFR (mL/min/1.73 m^2^) from baseline to two years after pemafibrate initiation. ^‡^ Log-transformed using the natural logarithm. ^§^ Changes in log-transformed parameters, calculated using the natural logarithm, from baseline to two years after pemafibrate initiation.

**Supplementary Table S4. Univariate linear regression models examining the association between two-year change in UACR and each clinical parameter**

| Dependent variables: ΔLog (UACR) ^†^ | | | |
| --- | --- | --- | --- |
| Independent variables | Unstandardized β | 95%CI | *P*-value |
| Clinical characteristics |  |  |  |
| Age | 0.015 | -0.005 to 0.034 | 0.136 |
| Female sex | -0.021 | -0.404 to 0.362 | 0.912 |
| Log (Duration of DM) ^‡^ | 0.050 | -0.140 to 0.240 | 0.601 |
| Current Smoker | 0.006 | -0.404 to 0.416 | 0.976 |
| BMI | -0.001 | -0.061 to 0.060 | 0.981 |
| Complications of DM |  |  |  |
| Neuropathy | 0.067 | -0.316 to 0.450 | 0.728 |
| Retinopathy | -0.438 | -0.822 to -0.053 | 0.026 |
| Atherosclerotic diseases | 0.114 | -0.329 to 0.557 | 0.610 |
| Physiological testing |  |  |  |
| SBP | -0.002 | -0.021 to 0.018 | 0.877 |
| DBP | -0.005 | -0.037 to 0.027 | 0.758 |
| Laboratory parameters |  |  |  |
| Log (HbA1c) ^‡^ | -2.080 | -3.702 to -0.459 | 0.013 |
| Log (TG) ^‡^ | -0.599 | -1.270 to 0.072 | 0.080 |
| ΔLog (TG) ^†^ | 0.828 | 0.490 to 1.167 | <0.001 |
| Log (HDL-C) ^‡^ | -0.472 | -1.654 to 0.709 | 0.429 |
| Log (LDL-C) ^‡^ | 0.152 | -1.326 to 1.629 | 0.839 |
| Log (AST) ^‡^ | -0.110 | -0.561 to 0.341 | 0.628 |
| Log (ALT) ^‡^ | -0.053 | -0.624 to 0.518 | 0.853 |
| Log (UA) ^‡^ | -0.351 | -0.916 to 0.214 | 0.220 |
| Log (UACR) ^‡^ | -0.335 | -0.477 to -0.192 | <0.001 |
| eGFR | 0.010 | -0.005 to 0.026 | 0.194 |
| hsCRP | -0.087 | -0.332 to 0.158 | 0.480 |
| Log (FGF21) ^‡^ | 0.208 | 0.011 to 0.405 | 0.039 |
| ΔLog (FGF21) ^†^ | 0.718 | 0.395 to 1.040 | <0.001 |
| Medications |  |  |  |
| Insulin | -0.320 | -0.795 to 0.156 | 0.185 |
| Sulfonylureas | -0.504 | -1.136 to 0.128 | 0.116 |
| Glinides | -0.146 | -0.787 to 0.495 | 0.651 |
| GLP-1 RAs | -0.064 | -0.457 to 0.330 | 0.748 |
| DPP4 inhibitors | -0.023 | -0.474 to 0.429 | 0.921 |
| Metformins | -0.133 | -0.518 to 0.253 | 0.495 |
| TZDs | -0.318 | -0.996 to 0.360 | 0.353 |
| Alpha-GIs | 0.038 | -0.757 to 0.834 | 0.924 |
| SGLT2 inhibitors | 0.095 | -0.289 to 0.479 | 0.624 |
| ARBs | -0.045 | -0.526 to 0.436 | 0.854 |
| MRAs | 0.073 | -0.397 to 0.543 | 0.759 |
| CCBs | 0.023 | -0.407 to 0.454 | 0.914 |
| Diuretics | 0.274 | -0.332 to 0.880 | 0.372 |
| Statins | 0.329 | -0.135 to 0.793 | 0.162 |
| UA lowering agents | 0.283 | -0.156 to 0.723 | 0.203 |

All independent variables, except for changes in parameters, corresponded to the values obtained at baseline. ALT, alanine transaminase; ARBs, angiotensin receptor blockers; AST, aspartate transaminase; BMI, body mass index; CCB, calcium channel blocker; CI, confidence interval; DBP, diastolic blood pressure; DM, diabetes mellitus; DPP4, dipeptidyl peptidase-4; eGFR, estimated glomerular filtration rate; FGF21, fibroblast growth factor 21; GIs, glycosidase inhibitors; GLP-1 RA, glucagon-like peptide-1 receptors agonist; HbA1c, hemoglobin A1c; HDL-C, high-density lipoprotein cholesterol; hsCRP, high-sensitivity C-reactive protein; LDL-C, low-density lipoprotein cholesterol; MRAs, mineralocorticoid receptor antagonists; β, regression coefficient; SBP, systolic blood pressure; SGLT2, sodium-glucose cotransporter 2; TG, triglycerides; TZDs, thiazolidinediones; T2DM, type 2 diabetes mellitus; UA, uric acid; UACR, urine albumin-to-creatinine ratio. ^†^ Change in log-transformed parameters, calculated using the natural logarithm, from baseline to two years after pemafibrate initiation. ^‡^ Log-transformed using the natural logarithm.

**Supplementary Table S5. Multivariate linear regression models examining the associations of baseline FGF21 levels and changes in FGF21 with the change in UACR**

| Dependent variable: ΔLog (UACR) ^†^ | | | | | | |
| --- | --- | --- | --- | --- | --- | --- |
| Independent  variables | Models excluding ΔLog (FGF21) | | | Models including ΔLog (FGF21) | | |
|  | Unstandardized β | 95%CI | *P*-value | Unstandardized β | 95%CI | *P*-value |
| Univariate model |  | | |  | | |
| Log (FGF21) ^‡^ | 0.208 | 0.011 to 0.405 | 0.039 | 0.208 | 0.011 to 0.405 | 0.039 |
| ΔLog (FGF21) ^†^ |  |  |  | 0.718 | 0.395 to 1.040 | <0.001 |
| Age and gender adjusted model | (Adjusted *R*^2^ = 0.033) | | | (Adjusted *R*^2^ = 0.192) | | |
| Log (FGF21) ^‡^ | 0.190 | 0.001 to 0.397 | 0.048 | 0.134 | -0.057 to 0.326 | 0.167 |
| ΔLog (FGF21) ^†^ |  |  |  | 0.678 | 0.341 to 1.015 | <0.001 |
| Age | 0.011 | -0.009 to 0.031 | 0.292 | 0.004 | -0.014 to 0.023 | 0.633 |
| Female sex | -0.118 | -0.501 to 0.265 | 0.542 | -0.194 | -0.546 to 0.159 | 0.277 |
| Liver enzymes adjusted model | (Adjusted *R*^2^ = 0.021) | | | (Adjusted *R*^2^ = 0.182) | | |
| Log (FGF21) ^‡^ | 0.212 | 0.012 to 0.412 | 0.038 | 0.134 | -0.053 to 0.320 | 0.158 |
| ΔLog (FGF21) ^†^ |  |  |  | 0.669 | 0.337 to 1.001 | <0.001 |
| Log (AST) ^‡^ | -0.178 | -0.745 to 0.390 | 0.535 | -0.172 | -0.691 to 0.347 | 0.511 |
| Log (ALT) ^‡^ | 0.106 | -0.612 to 0.823 | 0.770 | 0.141 | -0.515 to 0.797 | 0.670 |
| Multivariate model 1 | (Adjusted *R*^2^ = 0.395) | | | (Adjusted *R*^2^ = 0.457) | | |
| Log (FGF21) ^‡^ | 0.359 | 0.189 to 0.530 | <0.001 | 0.300 | 0.134 to 0.466 | <0.001 |
| ΔLog (FGF21) ^†^ |  |  |  | 0.445 | 0.162 to 0.727 | 0.002 |
| Age | 0.016 | 0.001 to 0.032 | 0.046 | 0.011 | -0.004 to 0.026 | 0.155 |
| Log (UACR) ^‡^ | -0.458 | -0.591 to  -0.324 | <0.001 | -0.408 | -0.539 to  -0.278 | <0.001 |
| Multivariate model 2 | (Adjusted *R*^2^ = 0.461) | | | (Adjusted *R*^2^ = 0.507) | | |
| Log (FGF21) ^‡^ | 0.445 | 0.265 to 0.624 | <0.001 | 0.389 | 0.212 to 0.565 | <0.001 |
| ΔLog (FGF21) ^†^ |  |  |  | 0.384 | 0.108 to 0.660 | 0.007 |
| Age | 0.008 | -0.008 to 0.024 | 0.308 | 0.005 | -0.010 to 0.021 | 0.500 |
| Log (UACR) ^‡^ | -0.461 | -0.593 to  -0.328 | <0.001 | -0.419 | -0.550 to  -0.289 | <0.001 |
| SBP | -0.013 | -0.028 to 0.002 | 0.090 | -0.014 | -0.028 to 0.001 | 0.059 |
| eGFR | 0.015 | 0.002 to 0.028 | 0.025 | 0.013 | 0.001 to 0.025 | 0.046 |
| Log (Duration of DM) ^‡^ | 0.071 | -0.078 to 0.219 | 0.345 | 0.044 | -0.099 to 0.187 | 0.545 |
| Log (HbA1c) ^‡^ | -1.201 | -2.465 to 0.062 | 0.062 | -1.038 | -2.253 to 0.177 | 0.093 |
| Multivariate model 3 | (Adjusted *R*^2^ = 0.435) | | | (Adjusted *R*^2^ = 0.545) | | |
| Log (FGF21) ^‡^ | 0.464 | 0.281 to 0.648 | <0.001 | 0.347 | 0.173 to 0.520 | <0.001 |
| ΔLog (FGF21) ^†^ |  |  |  | 0.328 | 0.056 to 0.601 | 0.019 |
| Age | 0.011 | -0.005 to 0.027 | 0.170 | 0.004 | -0.011 to 0.019 | 0.608 |
| Log (UACR) ^‡^ | -0.465 | -0.602 to  -0.328 | <0.001 | -0.395 | -0.522 to  -0.267 | <0.001 |
| SBP | -0.013 | -0.029 to 0.002 | 0.085 | -0.011 | -0.025 to 0.002 | 0.106 |
| eGFR | 0.014 | 0.001 to 0.028 | 0.036 | 0.008 | -0.004 to 0.020 | 0.201 |
| Log (TG) ^‡^ | -0.087 | -0.627 to 0.453 | 0.749 | -0.092 | -0.586 to 0.402 | 0.710 |
| ΔLog (TG) ^†§^ |  |  |  | 0.446 | 0.151 to 0.742 | 0.004 |

A multivariate linear regression analysis with crude and adjusted beta coefficients. All independent variables, except for changes in parameters, corresponded to the values obtained at baseline. ALT, alanine transaminase; AST, aspartate transaminase; CI, confidence interval; DM, diabetes mellitus; eGFR, estimated glomerular filtration rate; FGF21, fibroblast growth factor 21; HbA1c, hemoglobin A1c; β, regression coefficient; SBP, systolic blood pressure; TG, triglycerides; UACR, urine albumin-to-creatinine ratio. ^†^ Change in log-transformed parameters, calculated using the natural logarithm, from baseline to two years after pemafibrate initiation. ^‡^ Log-transformed using the natural logarithm. ^§^ Entered only into the models including changes in log-transformed FGF21 levels.
